# Assessing historical residential radon exposure using a glass-based surface trap method in low radon level areas: a pilot study

**DOI:** 10.1088/1361-6498/ae757a

**Published:** 2026-06-11

**Authors:** Ka Kahe, Denis L Henshaw, Yijia Zhang, Meghan Angley, Whitney Booker, Norman Kleiman

**Affiliations:** 1 Department of Obstetrics and Gynecology, Vagelos College of Physicians and Surgeons, Columbia University, New York, NY, United States of America; 2 Department of Epidemiology, Mailman School of Public Health, Columbia University, New York, NY, United States of America; 3 Atmospheric Chemistry Group, School of Chemistry, University of Bristol, Bristol, United Kingdom; 4 Department of Environmental Health Sciences, Mailman School of Public Health, Columbia University, New York, NY, United States of America

**Keywords:** radon, radon decay product, residential exposure assessment, glass-based surface trap method, epidemiology

## Abstract

Retrospectively estimating residential radon exposure in epidemiological studies is challenging using conventional methods. This study aimed to compare a modified glass-based surface trap technique for assessing historical indoor radon exposure with conventional long-term radon detectors among pregnant women from areas with relatively low radon levels. Ten women during their first trimester pregnancy were recruited from a Columbia University Irving Medical Center—affiliated hospital. Each participant was asked to place one detector in the basement and another in a high-occupancy main-floor room until delivery, after which devices were returned by prepaid mail to a certified laboratory. In addition, we collected the cover glass from small picture-frames that participants placed in the main-floor living area of their current and previous homes. Each glass plate was mounted against TASTRAK alpha-particle-detecting plastic and stored at—20 °C in a radon-proof atmosphere for 37 d. Plastics were etched and analysed for alpha-particle track density over a 4 cm^2^ area. Conventional detector measurements of indoor radon ranged from <14.8–133.2 Bq m^−3^ (<0.4–3.6 pCi l^-1^), while the modified glass-based method yielded 641–1476 tracks (0.14–0.32 Bq m^−2^). Spearman correlation (*ρ*) between these two measurements were 0.68 (main floor), 0.38 (basement), and 0.63 (average of both floors). After adjusting for detector exposure duration, the correlations were slightly increased to 0.72, 0.43, and 0.67, respectively. The modified glass-based surface trap method by assessing alpha-particle track counts demonstrated moderate-to-strong correlations with long-term radon detectors. While larger-scale studies are needed to confirm the findings, this technique may provide a practical alternative for reconstructing historical residential radon exposure, even in regions with relatively low radon levels.

## 1. Introduction

For decades, radon exposure has been recognised as a leading cause of lung cancer in addition to cigarette smoking (Darby *et al* 2005, World Health Organization 2009, The Conference of Radiation Control Program Directors [CRCPD] 2018). After inhalation, radon can distribute throughout the body according to its solubility and the integrity of the lung’s epithelial lining, reaching major organs in a complex combination of radon and its decay products (Richardson *et al* 1991, Parent 2005, Sakoda *et al* 2010). Radon exposure and tobacco smoke share certain pathological mechanisms (Alavanja 2002, Bonner *et al* 2006), and smoking itself has been linked to numerous chronic diseases. Despite this, most prior studies have focused primarily on radon’s contribution to lung cancer risk (National Research Council (US) Committee on Health Risks of Exposure to Radon 1999, Alavanja 2002, Bonner *et al* 2006). Emerging evidence indicates that radon exposure may also be associated with a range of other health outcomes, including stroke (Zhang *et al* 2023, Buchheit *et al* 2024), cardiovascular disease (Dong *et al* 2022), leucaemia (Ngoc *et al* 2022, Bozigar *et al* 2024, Chen 2024), melanoma (Boz *et al* 2024), mental health (Taylor *et al* 2024), hypertensive disorder in pregnancy (Papatheodorou *et al* 2021), and gestational diabetes (Zhang *et al* 2025). Additional epidemiological research is urgently needed to clarify these associations and strengthen causal inference, particularly given that randomised clinical trials are unlikely to be feasible.

A major challenge in radon research is accurately estimating historical or cumulative exposure at the individual level. Much of the existing literature relies on area-level estimates, such as county or zip code averages (Lu *et al* 2022, Zhang *et al* 2022, Angley *et al* 2024). While advanced statistical models may extrapolate these data to finer geographic scales (Li *et al* 2021), the resulting measurement error may introduce bias or confounding. Such estimates are useful for hypothesis generation, but individual-level residential data are essential for precise exposure assessment, especially as location and age of individual dwellings are associated with radon levels (Hahn *et al* 2023). Currently, the most common tools for assessing individual-level residential radon in large epidemiological studies are detectors, including short-term monitors (several days) and long-term solid-state nuclear track monitors (three months to a year) (Takahashi *et al* 2022, Grapentin *et al* 2024). Long-term detectors capture seasonal variation more effectively than short-term devices, but they cannot be used retrospectively to measure historical exposure across multiple residences.

**Figure 1.**
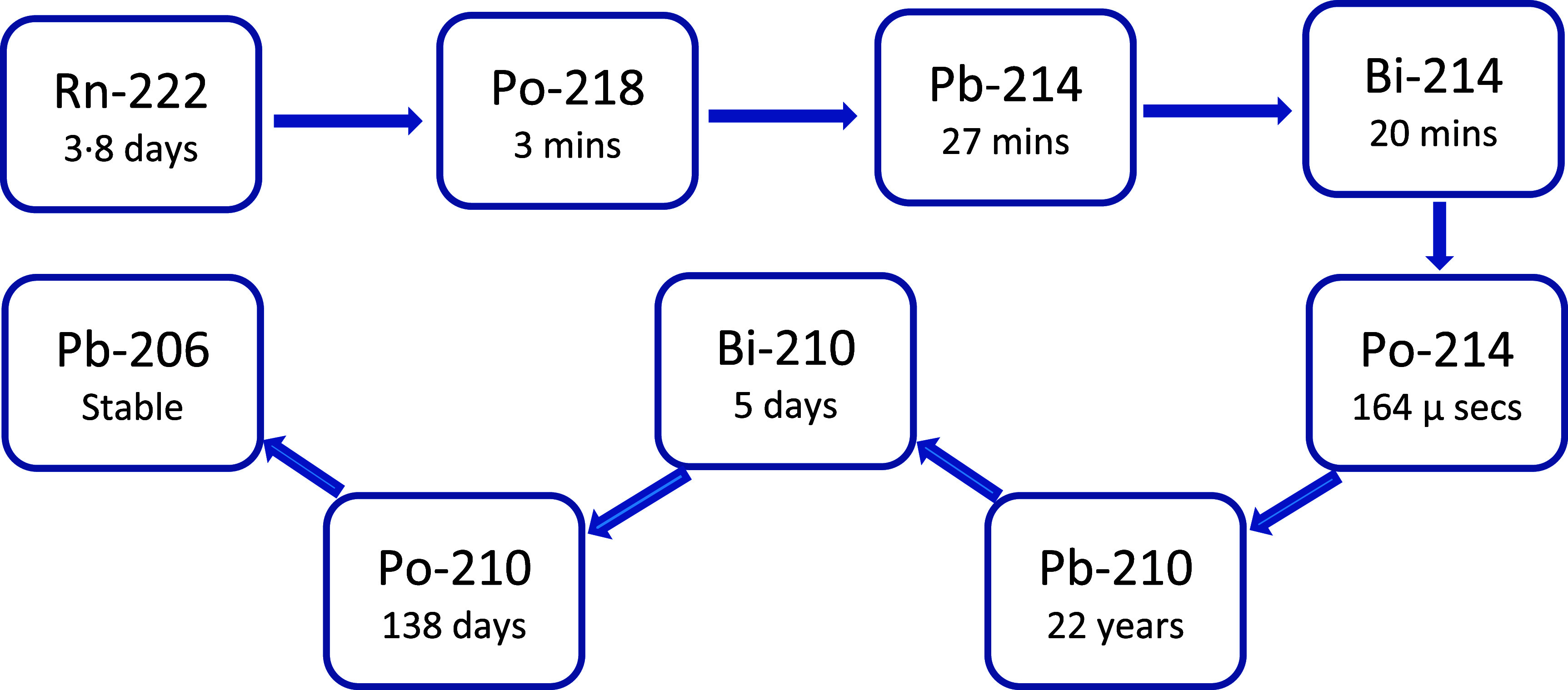
The predominant pathways in the decay of radon (Rn-222) and half-life down to its stable descendent.

To address this limitation, researchers have developed a surface trap technique (Samuelsson 1988, Weinberg 1995, Birovljev *et al* 2001). The decay chain for radon (Rn-222) includes the short-lived isotopes polonium-218 (Po-218), lead-214 (Pb-214), bismuth-214 (Bi-214), and polonium-214 (Po-214), which further decay into the longer-lived products lead-210 (Pb-210) and polonium-210 (Po-210) (figure 1) (Persson and Holm 2011). In air, the radon decay product Po-214 can deposit onto surfaces such as glass, leading to the incorporation of its decay product Pb-210 within a layer up to 0.1 *μ*m beneath the glass surface (Samuelsson 1988). Given its half-life of more than two decades, Pb-210 gradually accumulates in glass, serving as a long-term indicator of radon exposure. The embedded Pb-210 can be quantified by detecting the 5.3 MeV alpha emissions from its subsequent decay product, Po-210. Glass items such as mirrors and picture frames, which often accompany individuals across different residences, are suitable for this assay (Samuelsson 1988, Weinberg 1995, Birovljev *et al* 2001).

This approach has been demonstrated in proof-of-principle studies using two types of plastic alpha-particle track detector, CR-39 and LR115 (Bochicchio *et al* 2003, Das and Deb 2022). However, both detectors relied on manual track counting and provided no information on the energy—and therefore potential identity—of individual alpha-particles and their parent radionuclides. Significant improvements in this approach can be achieved by exploiting the spectroscopic features of alpha-particle measurements in CR-39 plastic, enabling estimation of individual alpha-particle energy (Fews and Henshaw 1982, Hatzialekou *et al* 1988).

This pilot study aimed to assess the feasibility of this modified glass-based surface trap method and generate preliminary data on the correlation between this method and contemporary long-term indoor radon detector assessments. The study design and protocol were approved by the Columbia University Irving Medical Center Institutional Review Board.

## 2. Materials/subjects and methods

### 2.1. Study design and participants

This pilot study was nested within a study designed to investigate the association between residential exposure to radon and PM_2.5_ and adverse pregnancy outcomes. Participants were recruited from New York Presbyterian Hospital at Columbia University Irving Medical Center. Ten pregnant women, aged 18 years or older, between 6^0/7^ and 13^6/7^ weeks of gestation, and residing in single-family homes, were enrolled. Each participant completed a brief questionnaire to collect sociodemographic, lifestyle, and residential information, e.g. education, income, and tobacco use (both conventional and electronic cigarettes). Participants also received two one-year indoor radon detectors—one for the basement and another for the main floor—along with detailed instructions on placement and return. Prior to their final prenatal visit, participants were reminded to mail the detectors to a certified radon laboratory using a prepaid envelope. Additionally, a small glass-surface picture frame, displayed on the main floor and carried over multiple households, was collected.

### 2.2. Glass-based surface trap measurement

**Figure 2.**
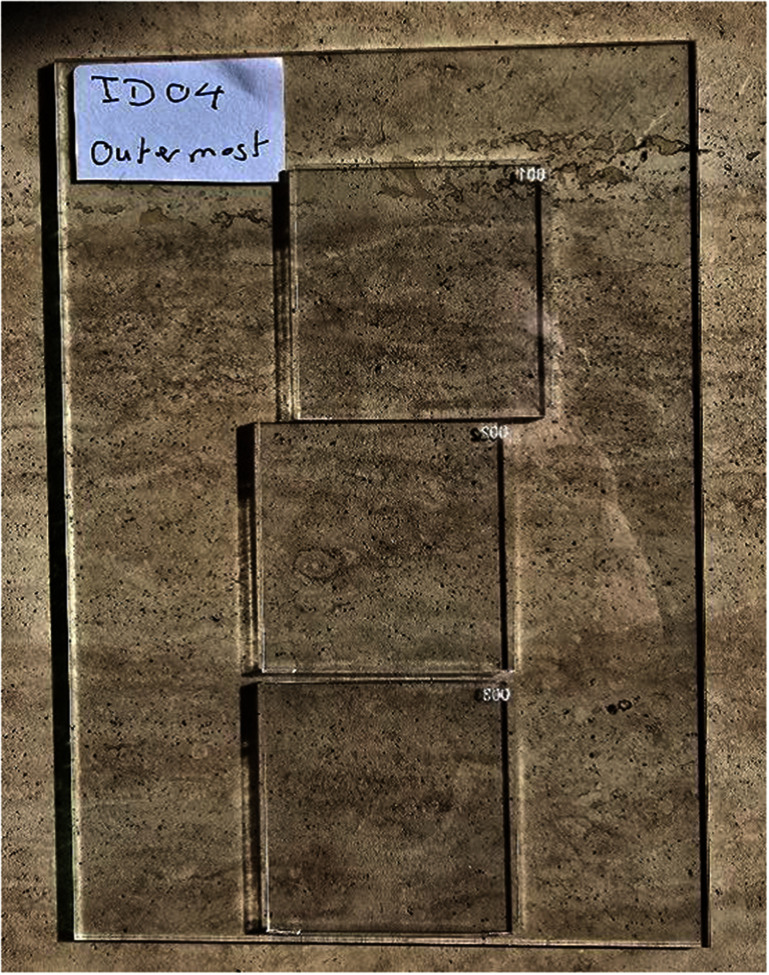
An example of how TASTRAK plastics mounted on the outer-facing surface.

CR-39 plastic detectors, marketed under the name TASTRAK were obtained from TASL, UK (TASL 2022). For each glass sample, three 5 × 5 cm TASTRAK plastics were mounted on the outer-facing surface: one for the primary exposure and two for backup. The side containing the TASTRAK ID code was placed in contact with the glass. Each sample was photographed to document proper placement, as illustrated in figure 2. The loaded samples were sealed individually in radon-proof metalized bags, placed in an additional metalized bag, and stored at −20 °C. After a 37 day exposure period, the TASTAK plastics were removed and immediately etched for one hour in 6.25 M NaOH at 98 °C. A 2 cm × 2 cm (4 cm^2^) area of each plastic was then scanned using the TASL*Image* scanning system (TASL 2022). The recorded area alpha-particle activity, *A* in units Bq m^-2^ is then related to the track count, *c* per cm^2^ via the relation:

\begin{equation*}A = \left( {c \times {{10}^{ - 4}}} \right)/\left( {d \times s} \right)\end{equation*}

where *d* = the number of days exposed and *s* = 24 × 3600, the number of seconds in a day. For a 37 day exposure, this corresponds to 1 count per cm^2^ = 3.1 × 10^−3^ Bq m^−2^.

The TASL*Image* software first isolates candidate alpha-particle etch tracks using a series of shape parameters and optical density. It then measures the parameters of each candidate track, including minor axis diameter and projected length (defined as the major axis diameter on steep tracks or the overall projected length for shallow tracks).

### 2.3. Long-term indoor detector assessment

Participants were instructed to place one AccuStar AT-100 alpha track device in a high-occupancy room on the main floor (i.e. ground level) and another in their basement. They were also asked to complete the information sheet that accompanies the detectors, which included recording the start and stop dates of the exposure period and the placement location (e.g. living room or bedroom). In addition, participants received a questionnaire, to be returned to the clinician or study office upon completion, that collected information on the acceptability of the detector placement and exposure duration, as well as personal spatial data (e.g. time spent at home, outdoors, or in other buildings) during the study period. Each returned detector was labelled with a unique personal identifier to ensure confidentiality of the information provided to the radon laboratory (Spruce Environmental Technologies, Inc.) (Radon.com 2026). The laboratory reported radon testing results to participants and investigators through a password-protected web portal, using unique detector identifiers.

### 2.4. Statistical analysis

Radon measurements were reported using two assessment methods (one-year detector and glass surface trap) as a median and range. To assess agreement between the methods, Spearman’s rank correlation coefficient (*ρ*) with corresponding *p*-values were calculated. This non-parametric approach was chosen because it does not assume linearity or normality and is less sensitive to outliers. Partial Spearman correlations were calculated by ranking the variables of interest and computing the partial Pearson correlation of the ranked residuals after adjusting for covariates (Iman and Conover 1979). For analysis, radon monitor values detected as <14.8 Bq m^−3^ (<0.4 pCi l^-1^) were assigned a value of 7.4 Bq m^−3^ (0.2 pCi l^-1^).

## 3. Results

All 10 participants lived in the tri-state (NY, NJ, CT) area in single-family homes with basements. On average, participants resided in their current homes for four years. The one-year detectors were deployed for a mean duration of 6.1 months (range: 4.7–7.4 months).

**Figure 3.**
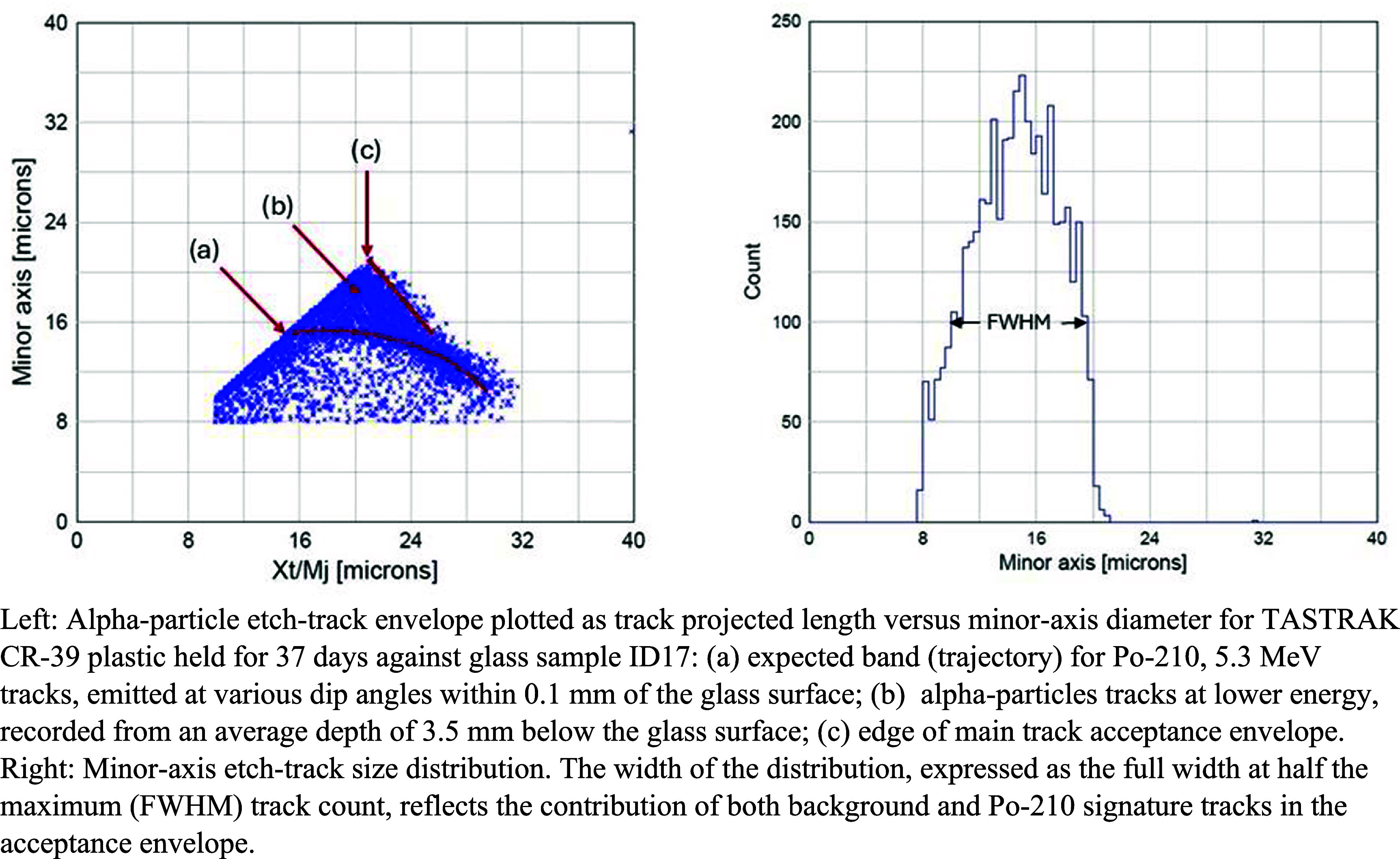
An illustration of expected band (trajectory) for Po-210 (5.3 MeV tracks) based on glass sample ID 17.

After 37 d of exposure against TASTRAK detectors, evidence of a distinct Po-210 signature was largely obscured by the high background activity inherent to the glass samples themselves. Notably, no characteristic peaks corresponding to the short-lived decay products Po-218 (6.0 MeV) and Po-214 (7.7 MeV) were observed, confirming that none of the plastic detectors were contaminated by airborne alpha activity (Fews *et al* 1999). These features are illustrated in figure 3, which presents the raw data for glass sample ID17. The presumed 5.3 Mev Po-210 band appears, as expected, close to the position of a 6.0 MeV Po-218 band (see figure 3 of Steck *et al* (2002)) (Fews *et al* 1999).

Because these candidate signals were buried within the background activity of the glass, we concluded that extracting a quantitative Po-210 measure would not be meaningful. Instead, we elected to use the total track count in the upper portion of the envelope, shown in the example in figure 3, for correlation with the long-term radon measurements.

Table 1 summarises radon assessments obtained using the glass surface trap method and long-term radon detectors. Alpha-particle tracks were counted over an area of 4 cm^2^ during a 37 day exposure period. The observed track densities ranged from 641 to 1476, corresponding to 0.50–1.15 Bq m^−2^. For the radon detectors, one basement measurement was missing. Median (range) radon concentrations were 42.6 (<14.8–96.2 Bq m^−3^) or 1.15 (<0.4–2.6 pCi l^-1^) on the main floor and 92.5 (14.8–133.2 Bq m^−3^) or 2.5 (0.4–3.6 pCi l^-1^) in the basement.

**Table 1.** Radon assessments by glass surface trap method and long-term radon detector (Bq m^–3^).

	Glass surface trap method		Long-term indoor radon detector		
Participants	Columbia glass #	Alpha-particle counts over 4 cm^2^	Main floor	Basement	Average
1	ID04	773	<14.8^a^	44.4	25.9
2	ID10	746	<14.8^a^	14.8	11.1
3	ID14	1476	81.4	118.4	99.9
4	ID16	1042	51.8	118.4	85.1
5	ID17	787	96.2	103.6	99.9
6	ID07	711	<14.8^a^	—^b^	7.4
7	ID12	726	51.8	133.2	92.5
8	ID15	773	37.0	62.9	50.0
9	ID18	641	<14.8^a^	62.9	35.2
10	ID11	1035	48.1	92.5	70.3

a If radon level <14.8 Bq m^−3^, 7.4 is assigned in the correlation analyses.

b Detector was missing.

The Spearman correlation coefficients were *ρ* = 0.68, *p*= 0.03 (glass vs main-floor detector), *ρ* = 0.38, *p*= 0.31 (glass vs basement detector), and *ρ* = 0.63, *p* = 0.049 (glass vs the average of both detectors). After adjusting for the exposure duration of each radon detector in the households, the Spearman correlation coefficients were strengthened. The corresponding correlations became 0.73 (*p* = 0.02), 0.43 (*p* = 0.28), and 0.67 (*p* = 0.048), respectively.

## 4. Discussion

In this pilot study, we demonstrated the feasibility of a modified glass-based surface trap method for estimating long-term radon exposure. We also examined its correlation with conventional indoor radon detector assessments over an average six-month exposure period in regions with relatively low radon levels. Preliminary results indicate moderate-to-strong correlations between the two measurement methods.

Specifically, the correlations (*ρ* = 38%–43%) between the glass sample and basement detectors appear moderate, likely for two reasons: (1) the glass surface picture frames were located on the main floor rather than in the basement, and (2) one of the ten basement detectors was missing. In contrast, we report a stronger correlation (*ρ* = 68%–73%) between the glass sample and main-floor detectors. Alpha-particle tracks on glass samples theoretically integrate cumulative radon exposure over decades, thereby including current and historical exposure across multiple residences. Consequently, the correlation may be attenuated by random variations in radon concentrations from prior residences. Hence, a perfect or very high correlation may not be expected.

The long-term radon detector remains the gold standard for assessing indoor radon exposure in epidemiological studies; however, it cannot capture exposures that occurred prior to their installation. In this study, the average detector placement period was slightly over six months rather than a full year. Nevertheless, previous research has shown that detectors placed across two seasons can provide results that approximate a one-year measurement (Field *et al* 1998). Findings from this pilot study suggest that the modified glass-based surface trap method by assessing alpha-particle track counts may serve as a viable alternative, offering sufficient information to rank participants by radon exposure for relative health risk estimation.

A few issues related to this glass-based measurement need to be acknowledged. We had limited success in detecting a distinct Po-210 signature from the glass samples. While some studies have reported correlations between Po-210 measurements in glass and contemporary radon levels—particularly in non-smoky environments—the findings have been inconsistent (Bochicchio *et al* 2003). This raises two considerations: why a clear Po-210 band was not observed in our glass samples, and what significance should be attributed to the total alpha-activity. Samuelsson (1988) originally proposed the glass-based method, detecting Po-210 derived from Pb-210 in a thin (up to 0.1 *μ*m) subsurface glass layer using a high-resolution pulse ionisation chamber. At lower energy resolution, however, which is inevitable here, the Po-210 peak may be obscured within the background alpha-activity, where *in-situ* ionisation chamber measurements are not practical for large-scale epidemiological applications.

In addition, previous investigators have attempted to assay surface Po-210 in glass using two types of plastic alpha-track detectors, CR-39 and LR115 (Falk *et al* 1996, Bochicchio *et al* 2003). CR-39 records alpha-particles across the full natural energy spectrum, whereas LR115 only detects particles with energies below 4.4 MeV. Since Po-210 emits alpha-particles at 5.3 MeV, LR115 fails to detect them at full energy, which may partially account for inconsistent findings in earlier studies (Nikezic and Yu 2002).

Moreover, as the heaviest noble gas, radon is capable of diffusing through non-conducting solids, though its behaviour in glass remains poorly characterised. If radon diffuses into glass and decays at depths greater than the commonly assumed 0.1 *µ*m Pb-210 surface layer, Pb-210 could become embedded more deeply. Subsequent alpha-decay of Po-210 might still be recorded by plastic track detectors, though at reduced energy compared to the full 5.3 MeV emission. One study has documented radon diffusion in glass (Arafa 2002), and such diffusion could explain the range of track sizes we observed without a clearer Po-210 band. In this scenario, much of what we classified as background may, in fact, represent a true Po-210 signal from accumulated Pb-210. Conventionally, background alpha activity in glass has been wholly attributed to uranium-238 (U-238) and thorium-232 (Th-232) decay series radionuclides, which should produce high-energy alphas such as 6.0 MeV Po-218, 7.7 MeV Po-214, and 8.8 MeV Po-212. Interestingly, no such long-range alpha tracks were detected in this study. These findings suggest that radon diffusion into glass may provide a meaningful signal for epidemiological use, and the radon diffusion constant in glass warrants further investigation in the context of Po-210 assessment. The findings also suggest that the width of the track minor axis size distribution is a useful indicator of the number of relevant recorded alpha-tracks. Notably, information on the duration of use of the glass samples (e.g. picture frames) and frequency with which they were cleaned or wiped was not collected, which might have introduced bias into our findings. Additionally, our assessments might be confounded by the lack of information on participants’ home ventilation characteristics, central air filtration, and other factors that could influence radon plate-out efficiency.

Furthermore, environmental tobacco smoke is another potential factor influencing Po-210 detection. Field *et al* noted that smoking may disrupt the correlation between radon and Po-210, presumably by reducing Pb-214 plate-out through attachment to smoke particles (1999). Bochicchio *et al* observed weak-to-moderate correlations between contemporary radon and Po-210 levels in non-smoky rooms, but no correlation in smoky environments (2003). In our pilot study, none of the participants were current smokers, but information on secondary smoke exposure was unavailable, which limits our ability to explore potential impacts of environmental tobacco use.

Several studies have evaluated the original glass-based method against contemporary radon detector measurements, primarily in areas with relatively high radon levels. Birovljev *et al* examined 17 dwellings in Norway, where the mean radon concentration was 3,703 Bq m^−3^, and reported a Pearson’s correlation of 0.877 (2001). Žunić *et al* assessed implanted Po-210 on glass surfaces in 32 Serbian homes with radon concentrations ranging from 1,040 to 1,380 Bq m^−3^, finding a correlation of 0.73 (2007). In Italy, Bochicchio *et al* investigated 20 dwellings with an average radon level of 133 Bq m^−3^ and observed a correlation of 0.67 between the glass surface method and conventional detectors. Notably, the correlation rose to 0.83 in non-smoking households, highlighting the influence of smoking (Bochicchio *et al* 2003). Similarly, Steck *et al* analysed implanted Po-210 in glass samples alongside year-long detector measurements from 24 homes in Iowa and Missouri with an average radon level of 177 Bq m^−3^, reporting correlations of 0.81 overall and 0.84 in non-smoking homes (2002). In contrast, an earlier study by Mahaffey *et al*, conducted among 19 households within the Missouri Women’s Health Study, found a modest correlation of 0.48 between CR-39 surface monitors affixed to glass, ceramic, or enamelled objects in the kitchen or bedroom and year-long radon exposure (1993).

Our study was conducted in regions with relatively low radon levels (average radon = 39.6 Bq m^−3^) and our findings were generally consistent with the prior studies. In the present study, we employed an analysis based on the TASL*Image* software used in the UK-approved TASL radon detectors using TASTRAK (PADC/CR-39). This software measured track size distributions consistent with those reported in prior work (Fews and Henshaw 1982, Hatzialekou *et al* 1988). In particular, the TASL*Image* software defines tracks size acceptance envelopes and uses these as a self-calibrating feature (Hatzialekou *et al* 1988). However, the algorithm is not specifically tuned for detecting Po-210 from glass, which is further complicated by the uncertain presence of a Po-210 band. For future studies, it may be advantageous to employ three plastics per glass sample, etched separately for different times, to map the potential evolution of a Po-210 band.

## 5. Conclusions

This pilot study demonstrates the feasibility and acceptability of a modified glass-based surface trap method that assesses alpha-particle track counts instead of Po-210 and shows moderate-to-strong correlations with long-term indoor radon detector measurements. Since the primary goal of epidemiological studies is to estimate relative health risk based on cumulative exposure, the glass-based approach appears suitable for ranking participants by exposure even in low radon areas, while uniquely enabling retrospective reconstruction of residential radon exposure—a capability not afforded by conventional detectors. Refining cumulative Po-210 detection from glass into a routine, cost-effective tool for historical radon assessment in regions with relatively low radon levels could hold substantial public health value.

## Data Availability

The data cannot be made publicly available upon publication because they contain sensitive personal information. The data that support the findings of this study are available upon reasonable request from the authors.
